# Identification and characterization of putative effectors from *Plasmodiophora brassicae* that suppress or induce cell death in *Nicotiana benthamiana*

**DOI:** 10.3389/fpls.2022.881992

**Published:** 2022-09-20

**Authors:** Zongxiang Zhan, Huishan Liu, Yao Yang, Shuang Liu, Xiaonan Li, Zhongyun Piao

**Affiliations:** College of Horticulture, Shenyang Agricultural University, Shenyang, Liaoning, China

**Keywords:** clubroot disease, *Plasmodiophora brassicae*, secretory proteins, plant immunity, effectors

## Abstract

Clubroot, caused by *Plasmodiophora brassicae*, is a major disease of crucifers. Effector proteins are important virulence factors in host recognition of pathogens and the interactions between pathogens and hosts. Secretory proteins, as effector candidates, have been studied in the interaction between *Plasmodiophora brassicae* and its hosts. In this study, 518 secretary proteins were screened from the *Plasmodiophora brassicae* genome. A total of 63 candidate effectors that induce or suppress cell death were identified using agroinfiltration-mediated transient expression in *Nicothiana benthamiana*. The candidate effectors, Pb4_102097 and Pb4_108104 showed high expressing level in the stage of rest spore maturity, could induce cell death and were associated with H_2_O_2_ accumulation in *N. benthamiana* leaves. In addition, 55 candidate effectors that could suppress BAX (Bcl-2-associated X protein) induced cell death, and 21 out of which could suppress the immunity caused by bacterial pathogen *Pseudomonas syringae* pv. tomato strain DC3000 expressing *avrRps4* in *Arabidopsis*. Based on the expression pattern in different stages, 28 candidate effectors showed high expression levels during the primary and secondary infection stage. Five candidate effectors containing the RXLR motif functioned in the cytoplasm and cell membrane.

## Introduction

Plants rely on innate immunity and the transmission of systemic signals from pathogen infection sites to resist pathogen invasion (Dangl and Jones, 2001; Chisholm et al., 2006; Jones and Dangl, 2006). Pathogens have conserved pathogen-associated molecular patterns (PAMPs), such as flagellin, that lead to PAMP-triggered immunity (PTI). Pathogens can secrete effector proteins targeted to plant apoplasts or delivered inside the host cytoplasm to suppress PTI and contribute to successful infection. Plants have an internal immune receptor-disease resistance gene (R-gene) encoding a nucleotide-binding leucine-rich repeat (NB-LRR) protein to recognize effectors and induce defense responses. These responses include programmed cell death (PCD) and reactive oxygen species (ROS) bursts and are known as effector-triggered immunity (ETI). *Plasmodiophora brassicae* is a well-adapted pathogen of *Brassica* but PTI and ETI are not well-characterized in the host-*P. brassicae* pathosystem (Pérez-López et al., 2018).

*P. brassicae* is an intracellular obligate biotrophic plant pathogen responsible for clubroot disease in the *Brassicaceae* (Kageyama and Asano, 2009). The life cycle of *P. brassicae* has three stages. In the soil survival stage, resting spores germinate to primary zoospores under optimal conditions. In the primary infection stage, primary zoospores colonize root hairs and form primary plasmodia and cleave into zoosporangia. Secondary zoospores released from zoosporangia penetrate the cortical tissues. The secondary infection develops into secondary plasmodia, which proliferate and produce characteristic swollen gall or club-shaped plant roots (Kageyama and Asano, 2009; Feng et al., 2012). The infection stages are critical periods for the host to recognize the pathogen and establish an immune defense system (McDonald et al., 2014). However, the recognition mechanism and interaction between *P. brassicae* and hosts harboring clubroot resistance genes is unknown.

To facilitate host plant colonization many pathogens secrete proteins and other molecules, collectively known as effectors, in the hosts to disable the plant defenses (Hogenhout et al., 2009). Many effectors have been characterized in bacteria, fungi, oomycetes and nematodes using agroinfiltration of *Nicotiana benthamiana* (Pitino et al., 2016). The development of bioinformatics and ultra-deep sequencing of plant pathogenic microbes has enabled genome-wide putative effectors genes to be cataloged based on special motifs (Huang et al., 2005; Liu et al., 2014). The basic criteria used to identify candidate secreted effector proteins include proteins with a signal peptide (within the initial 60 amino acids at the N-terminus), no trans-membrane domains and a size of 300 to 450 amino acids (Pérez-López et al., 2020). Many predicted effectors in each pathogen can be identified using bioinformatics analysis but assessing the functions of the candidate effectors is challenging. In the combined secretome of *Plasmopara viticola*, 51 predicted effectors with the RXLR motif were identified and 23 fully suppressed programmed cell death that was elicited by cell death-inducing proteins in *N. benthamiana* (Ling et al., 2015; Xiang et al., 2016). In fungi, 18 of 30 randomly selected putative effectors of *Ustilaginoidea virens* suppressed the *Burkholderia glum*ae-triggered hypersensitive reaction (HR) at different levels in *N. benthamiana* (Zhang et al., 2014). In the cereal cyst nematode, *Heterodera avenae*, 95 candidate effector genes were evaluated and 78 effectors suppressed BT-PCD in *N. benthamiana* leaves (Lin et al., 2016).

A limited number of *P. brassicae* effectors have been functionally characterized based on the genome of sequences (Rolfe et al., 2016). A total of 553 secreted proteins in *P. brassicae* isolate e3 were predicted by bioinformatics analysis. A secretory methyltransferase can methylate salicylic acid and alter host susceptibility to *P. brassicae* (Schwelm et al., 2015). During primary infection, 23 of 33 secretory proteins suppressed mouse Bcl-2-associated X protein (BAX)-induced cell death and two effectors could induce cell death in *N. benthamiana* (Chen et al., 2019). And endomembrane-targeting *P. brassicae* effectors were reported to modulate PAMP and triggered immune responses in plants (Hossain et al., 2021). However, the other putative effectors at different infection stages and isolates have not been identified and characterized.

In this study, secreted protein genes were initially screened and predicted by bioinformatics software. A total of 63 candidate effectors of *P. brassicae* were selected and cloned. The capacities of these secretory proteins to induce or suppress cell death were assessed. The results of these experiments helped to reveal the pathogenic mechanism of *P. brassicae* and its interactions with host plants.

## Materials and methods

### Plant materials, *Plasmodiophora brassicae* isolate, and culture conditions

*Nicotiana benthamiana* and susceptible Chinese cabbage ‘91–12’ (*Brassica rapa*) were grown at 24°C and a 16:8 h (L:D) photoperiod. *N. benthamiana* was used for transient agro-Infiltration assays. The susceptible ‘91–12’ was inoculated with *P. brassicae* and the infected root was collected for *P. brassicae* RNA extraction. The *P. brassicae* isolate used in this study was collected from a *B. rapa* field located in Xinmin, Liaoning Province, China. This pathotype was Pb4 based on the result of the Williams system. The club roots were stored at −80°C until used.

### Bioinformatic identification of *Plasmodiophora brassicae* secreted effector candidates

The putative effector genes were mined from the genome sequence of *P. brassicae* (PRJNA851541). The candidate secreted effector proteins were screened according to the basic criteria: proteins with a signal peptide, no trans-membrane domains and size less than 450 amino acids. The N-terminal signal peptides were predicted using SignalP 4.1^1^ with Default D-cutoff values (Petersen et al., 2011). The proteins with a transmembrane domain or mitochondrial signal peptides were removed after analyzing with TMHMM and TargetP (Krogh et al., 2001; Emanuelsson et al., 2007), respectively. Proteins less than 450 amino acids, with signal peptides but lacking any transmembrane domains were regarded as candidate secreted effectors. The PFAM^2^ (Finn et al., 2014) online software was employed to characterize the candidate effector domains of *P. brassicae*.

### RNA extraction, cDNA synthesis, and RT-PCR expression analysis

Tissue samples from both *P. brassicae* inoculated or non-inoculated *B. rapa* ‘91–12’ roots at 5, 14, 21 and 28 DPI (days post inoculation) were collected in liquid nitrogen. Total RNA was extracted using Monzol (Monad, SuZhou, China). cDNA synthesis was carried out using MonScript™ RTIII All-in-One Mix with dsDNase kit (Monad, SuZhou, China) following manufacturer instructions. RT-PCR was performed using SuperReal PreMix Plus (SYBR Green) in a 20 μl final volume. Information on primers is provided in Supplementary Table S1. GraphPad Prism (Swift, 1997) software was employed to construct the heat map based on the expression pattern in different stages.

### RNA extraction and plasmid construction

RNA were extracted from *P. brassicae*-infected roots using the TRIzol reagent (Invitrogen, Carlsbad, CA, United States) according to the manufacturer’s recommended protocol. First-strand cDNA was synthesized using an RT-PCR system (Promega, Madison, WI, United States) following the manufacturer’s instructions. Primers were designed according to the instructions on the In-Fusion® HD Cloning kit based on the coding sequence in Supplementary Table S2. The PCR amplifications were purified with TIANGEN DNA purification kit (DP214), and cloned to the vector pGR106. BAX and GFP genes were also cloned to vector pGR106 according to the method described above.

### Subcellular localization

The PCR-generated open reading frame of the candidate effector genes without stop codons was cloned in-frame upstream of the GFP gene in the binary potato virus X (PVX) vector pGR106-GFP. The constructed vectors were transformed in *Agrobacterium* GV3101 and cultivated with LB medium supplemented with 50 μg ml^−1^ of rifampicin and 50 μg ml^−1^ kanamycin and then used to transform *N. benthamiana* for transient expression studies. GFP signal was detected at room temperature after 24 h of expression using confocal fluorescence microscopy (Zeiss, LSM510 Meta, Carl Zeiss Germany).

### Agrobacterium co-infiltration and cell death assay

Agroinfiltration-mediated transient expression in *N. benthamiana* was performed as described by Bourras et al. (2015). The constructed vectors connected with the putative effector ORF sequence (with signal peptide) were transformed into *Agrobacterium tumefaciens* strain GV3101. To identify the putative effectors that induced cell death or suppressed cell death triggered by BAX, the OD_600_ value of the *A. tumefaciens* suspension was adjusted to 0.4, and *A. tumefaciens* carrying pGR106-BAX was injected 12 h after the injection of *A. tumefaciens* carrying putative effector genes. The phenotypic character of injection sites was observed after 4–5 days of agro-infiltration. *Agrobacterium* cultures carrying pGR106-GFP or pGR106-BAX were infiltrated in parallel as controls.

### Detection of H_2_O_2_ accumulation in the injection sites

To clarify the accumulation of reactive oxygen species (ROS) induced by the candidate effectors in the process of *Agrobacterium* inducing *N. benthamiana* leaves (Thordal-Christensen et al., 1997). The accumulation of H_2_O_2_ was detected by 3,3′-diaminobenzidine (DAB). The *N. benthamiana* leaves were harvested at 3 DPI with *A. tumefaciens*, and soaked in 1 mg/ml DAB solution at room temperature for 3 days. Then, the leaves were discolored by boiling in 99.9% ethanol for 25 min, and transparency with saturated trichloroacetic aldehyde solution and photographed.

### *Plasmodiophora Syringae* inoculation infection

The *P. syringae* strain Pst DC3000 (*AvrRps4*) was cultivated in King’s B medium containing rifampicin (50 μg/ml). After overnight culture with shaking, the bacteria were harvested by centrifugation at 4,000 rpm/min, washed, resuspended in 10 mM MgCl_2_, and diluted to the required density. *Pst* DC3000 (*AvrRps4*) was hand-infiltrated into the abaxial air space of the 4-week-old leaves 12 h after the injection of *A. tumefaciens* carrying putative effector genes or GFP as control. And the seedlings were cultured for 3 days for evaluate the bacterial populations. Six leaves were selected for evaluate for bacterial disease assay. And the methods followed the procedures described by Yuan and Xin (2021).

## Results

### Sixty-three putative *Plasmodiophora brassicae* effectors were selected

The genomic DNA of rest spore DNA from *P. brassicae* strain used in this study was extracted and re-sequenced with PacBio technic. The 25.25 MB genome was *de novo* assembled into 28 scaffolds with an N50 size of 1.23 MB. Total 13,036 genes were predicted with Augustus software (PRJNA851541). In order to mine the putative effectors, all the annotated genes were screened with SignalP 4.1, TMHMM and TargetP software. A total of 518 secreted proteins with a signal peptide with no trans-membrane domains and a size of less than 450 amino acids were identified and assigned as candidate effectors (Supplementary Table S2). And 449 proteins showed high similarity with the annotated proteins of *P. brassicae*. Another 17 proteins were homologous with other organism, such as *Pseudomonas*, *Chlorobium*, and *Acanthamoeba castellanii*. The left proteins were probably *P. brassicae*-specific, which have no assigned function or information in NCBI database (Supplementary Table S2).

Among these possible effectors, 225 had annotated domains, including Chitin-binding domain, Leucine Rich repeats Pkinase domain after blast against the pfam database (Supplementary Table S3). The number of candidate effectors containing the RXLR, LXAR, and LXLFLAK motifs were identified as effectors in some oomycetes and fungi was 28, 11, and 12, respectively (Supplementary Table S3). Considering the special character of *P. brassicae*, total 63 candidate effectors with different domains, containing an RXLR, LXAR, LXLFLAK motif or even only signal peptide were broad selected for further experimental verification (Table 1).

**Table 1:** Details of putative effector proteins.

Proteins	Functional domain	Amino acid	Homogeneous protein accession	*E*-value	Induce cell death	Suppress cell death
Pb4_108188	LFAFLAG	198	CEO96199.1	2E-137	NO	9/12
Pb4_111396	LMAR	230	CEP01277.1	2E-152	NO	11/12
Pb4_108186	LNAR	284	No significant similarity found.	/	NO	8/12
Pb4_103428	LNAR	322	CEO96521.1	0	NO	9/12
Pb4_111952	LYAR	139	SPQ96152.1	2E-90	NO	8/12
Pb4_102877	RHLR	219	CEP02928.1	3E-116	NO	11/12
Pb4_103507	RPLR	152	CEO98480.1	1E-95	NO	8/12
Pb4_108519	RQLR	226	CEO98814.1	8E-137	NO	9/12
Pb4_105958	RRLR	145	CEO97274.1	8E-46	NO	8/12
Pb4_100248	RRLR	220	No significant similarity found.	/	NO	9/12
Pb4_111854	RWLR	256	CEO94571.1	2E-163	NO	11/12
Pb4_104154	IRRFLAK	155	SPQ95396.1	2E-69	NO	10/12
Pb4_110819	Ankyrin repeats (3 copies)	262	QGW67320.1	6E-168	NO	8/12
Pb4_102521	Ankyrin repeats (3 copies)	185	CEO98859.1	2E-126	NO	10/12
Pb4_100008	Chitin recognition protein	223	CEO94946.1	1E-65	NO	9/12
Pb4_104095	Chitin recognition protein	173	CEP03198.1	3E-64	NO	8/12
Pb4_111366	Chitin recognition protein	132	CEP01301.1	6E-91	NO	11/12
Pb4_110950	Cysteine-rich secretory protein family	209	CEP02527.1	4E-117	NO	8/12
Pb4_108104	Cysteine-rich secretory protein family	159	SPQ99197.1	2E-35	13/15	/
Pb4_100099	Dopa 4,5-dioxygenase family	172	CEO95011.1	7E-109	NO	8/12
Pb4_105844	Eukaryotic-type carbonic anhydrase	293	CEO97180.1	0	NO	9/12
Pb4_112448	Fasciclin domain	163	CEP01688.1	3E-105	NO	11/12
Pb4_111688	Kazal-type serine protease inhibitor domain	184	CEO94444.1	2E-83	NO	8/12
Pb4_104979	Kazal-type serine protease inhibitor domain	177	CEO99342.1	2E-114	NO	8/12
Pb4_110334	Leucine rich repeat	239	CEO99250.1	2E-145	NO	9/12
Pb4_108437	Leucine rich repeat	207	CEO98761.1	9E-127	NO	10/12
Pb4_105970	Leucine Rich repeats (2 copies)	287	CEO97282.1	0	NO	9/12
Pb4_108273	MORN repeat	249	CEO98701.1	3E-70	NO	10/12
Pb4_108279	MORN repeat	252	CEP00030.1	5E-159	NO	8/12
Pb4_109192	Pam16	126	CEO95852.1	1E-56	NO	9/12
Pb4_109755	Phage Tail Collar Domain	191	CEO97090.1	6E-123	NO	8/12
Pb4_106798	Polysaccharide deacetylase	263	CEP01965.1	0	NO	10/12
Pb4_103612	Protein kinase domain	311	CEO98550.1	0	NO	10/12
Pb4_112853	Ras family	232	CEO94295.1	1E-153	NO	8/12
Pb4_111752	RCLR	221	CEO94501.1	3E-143	NO	10/12
Pb4_109724	Reeler domain	167	CEO97066.1	3E-121	NO	8/12
Pb4_100510	S1/P1 Nuclease	290	CEO95221.1	0	NO	9/12
Pb4_104106	Sep15/SelM redox domain	149	CEP03187.1	2E-84	NO	10/12
Pb4_107359	Tetrapyrrole (Corrin/Porphyrin) Methylases	285	CEO98691.1	0	NO	8/12
Pb4_106909	Thaumatin family	240	CEO94762.1	1E-173	NO	8/12
Pb4_106616	Thaumatin family	184	CEP00143.1	2E-176	NO	9/12
Pb4_106352	TLR4 regulator and MIR-interacting MSAP	190	CEO97603.1	7E-101	NO	8/12
Pb4_101162	NO	176	CEP00427.1	1E-126	NO	8/12
Pb4_107662	NO	165	CEO96817.1	5E-115	NO	9/12
Pb4_111336	NO	324	CEP01326.1	0	NO	8/12
Pb4_106019	NO	215	CEO97326.1	1E-146	NO	10/12
Pb4_103799	NO	146	CEP03452.1	6E-102	NO	8/12
Pb4_104606	NO	129	CEP03661.1	5E-51	NO	8/12
Pb4_106058	NO	232	CEO97355.1	2E-159	NO	8/12
Pb4_105542	NO	97	CEO97706.1	5E-48	NO	9/12
Pb4_112714	NO	213	CEP03506.1	8E-73	NO	10/12
Pb4_109005	NO	254	CEP02988.1	0	NO	10/12
Pb4_107465	NO	212	CEO98641.1	7E-74	NO	10/12
Pb4_102097	NO	277	CEP01497.1	0	12/15	/
Pb4_111712	NO	289	CEO94465.1	2E-135	NO	8/12
Pb4_109599	NO	204	CEP00938.1	1E-133	NO	9/12
Pb4_107399	NO	311	CEO98689.1	3E-177	NO	8/12
Pb4_110503	NO	290	CEO96920.1	0	NO	8/12
Pb4_103566	NO	74	CEO98520.1	2E-13	NO	10/12
Pb4_103257	NO	104	CEO96644.1	2E-21	NO	9/12
Pb4_108346	NO	145	CEO99975.1	7E-59	NO	10/12
Pb4_103736	NO	163	CEO98965.1	1E-106	NO	10/12
Pb4_105361	NO	137	CEO97828.1	6E-94	NO	10/12

### Two candidate effectors can induce cell death and H_2_O_2_ accumulation in *Nicothiana benthamiana*

**Figure 1:**
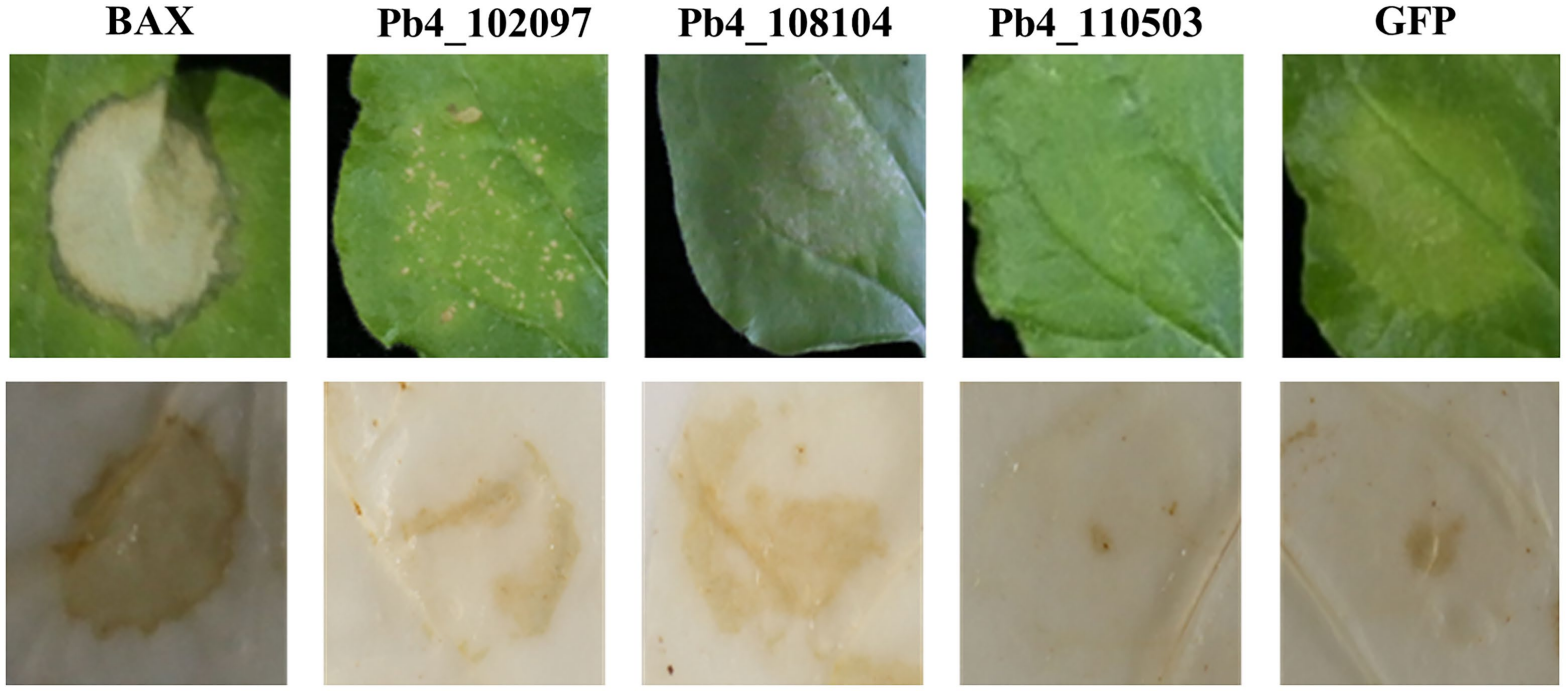
Putative effectors of *Plasmodiophora brassicae* induced programmed cell death and H_2_O_2_ accumulation in *Nicothiana benthamiana*. Necrosis and H_2_O_2_ accumulation in *N. benthamiana* leaves expressing BAX, Pb4_102097, Pb4_108104, Pb4_110503, and GFP (from left to right). HR cell death were observed four to five days after the infiltration.

To determine if any of the 63 proteins could induce cell death, all the candidate effector genes were transiently expressed in the leaves of *N. benthamiana* with the *Agrobacterium*-mediated plant virus transient expression method. Leaf necrosis was observed at 7 DPI. Necrosis was only found on the *N. benthamiana* leaves expressing the candidate effectors encoded by the *Pb4_102097* and *Pb4_108104* genes but not in other putative effectors, et *Pb4_110503* (Figure 1; Supplementary Figure S1). These data indicated that the two candidate effectors could strongly activate the immune system of *N. benthamiana*. However, the degree of cell death was lower than the positive control (leaves expressing BAX), which indicated that the proteins encoded by these two genes induced cell death in *N. benthamiana* less effectively than BAX Although a slight yellowing phenotype was found in negative control (leaves expressing eGFP), no obvious necrosis was found.

Cell death induced by effectors is often associated with H_2_O_2_ accumulation. *N. benthamiana* leaves expressing *Pb4_102097*, *Pb4_108104*, *Pb4_110503*, *BAX*, and *eGFP* were harvested for DAB staining. Brown substances showed that H_2_O_2_ accumulation was present in leaves expressing *Pb4_102097*, *Pb4_108104*, and *BAX* but not in leaves expressing *eGFP* and *Pb4_110503* (Figure 1). Leaves expressing *Pb4_102097*, *Pb4_108104* were stained a light color compared with leaves expressing BAX. These results demonstrated that both *Pb4_102097* and *Pb4_108104* could induce cell death and produce H_2_O_2_ accumulation.

### Twenty-one candidate effectors suppressed the immunity induced by BAX in *Nicothiana benthamiana* and avrRps4 in *Arabidopsis*

**Figure 2:**
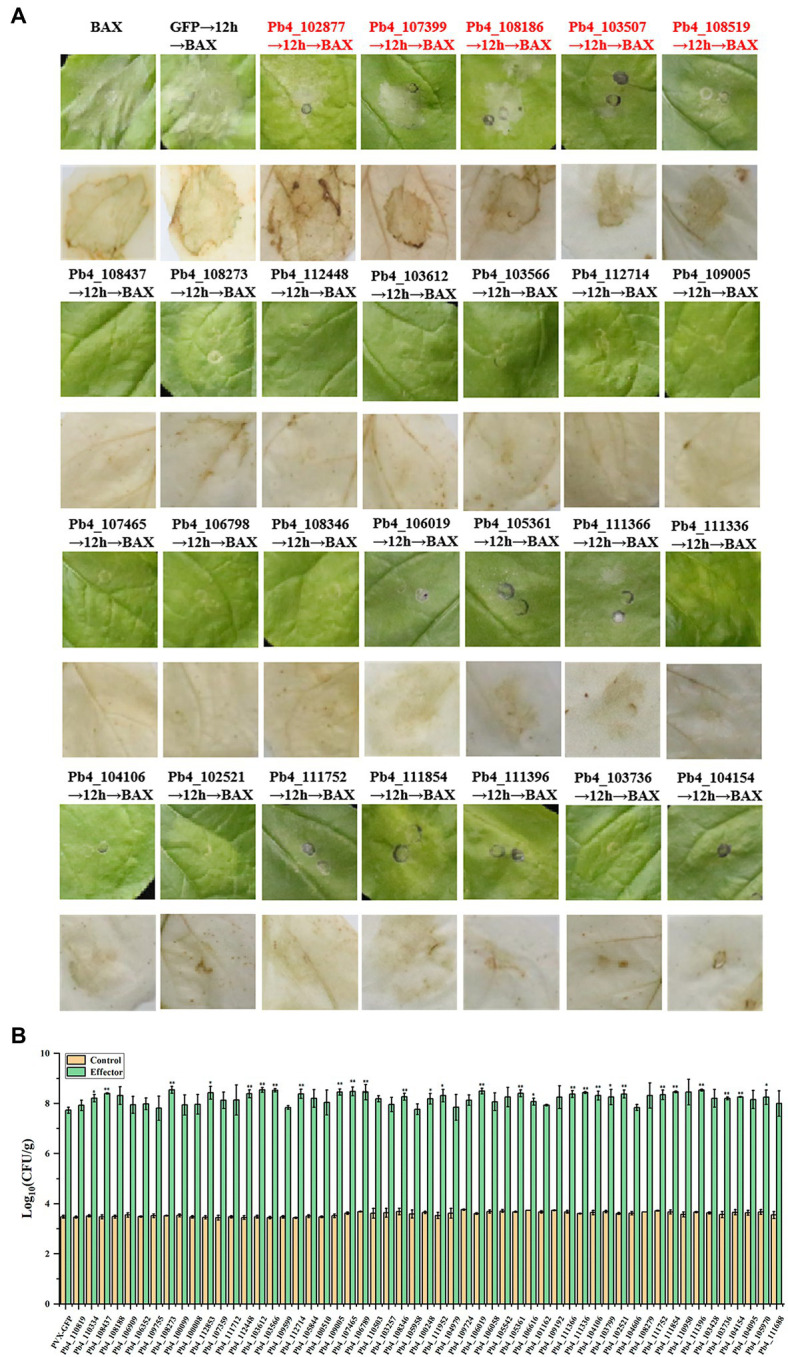
Analysis the putative effectors function in suppressing the immunoreaction in *Nicothiana benthamiana* and Arabidopsis. **(A)** Symptoms of transient expression of putative effectors in *N. benthamiana* leaves. The red font were the effectors which could not suppress BAX-induced cell death. The black font were the effectors significantly suppress BAX-induced cell death. HR cell death were observed four to five days after the infiltration. **(B)** Bacterial growth quantification of *Pst DC3000* (AvrRps4) in the *Arabidopsis* leaves expressing the putative effector. 4-week-old plants were infiltrated with OD600 = 0.0001 after the injection of *Agrobacterium tumefaciens* carrying putative effector genes or GFP as control and the samples were collected at 0 (yellow bars) and 3 dpi (green bars) for assay. Error bars represent SD of the mean of six samples. Significance difference between treatment and control groups (*t*-test): *p < 0.05; **p < 0.01.

To identify all the remaining 61 effectors’ possible roles in suppressing plant immunity, *A. tumefaciens* carrying pGR106-BAX was injected 12 h after the injection of *A. tumefaciens* carrying putative effector genes. Then, the presence of necrosis on *N. benthamiana* leaves was observed and the accumulation of H_2_O_2_ was determined. Severe necrosis was observed on the positive control leaves, which were injected with *Agrobacterium* carrying pGR-GFP and pGR-BAX, respectively. Necrosis was observed only in *N. benthamiana* leaves expressing Pb4_102877, Pb4_107399, Pb4_108186, Pb4_103507, and Pb4_108519 (Figure 2A). However, the degree of cell death and accumulation of H_2_O_2_ in the leaves expressing Pb4_103507, Pb4_108519 were lower than the positive control (Figure 2A). In contrast, the level of cell death of Pb4_102877, Pb4_107399, and Pb4_108186 were greater than the positive control (Figure 2A). The accumulation of H_2_O_2_ was also greater than the positive control. This result indicated that these putative effectors above could not inhibit the plant immunity induced by BAX (Figure 2A). However, 55 putative effectors could strongly suppress the cell death and accumulation of H_2_O_2_ induced by BAX, such as Pb4_108437 and Pb4_108237 (Figure 2A; Supplementary Figure S2).

In order to confirm the responsibility of these 55 putative effectors to plant immunity, bacteria growth assay was explored with Pst DC3000 (*AvrRps4*) in *Arabidopsis* leaves. The amount of bacteria growth in the leaves expression the 28 putative effectors were higher than that of control (*p* < 0.05), 21 of 28 showed significant difference (*p* < 0.01, Figure 2B; Supplementary Figure S3). However, there were no significant in the number of bacteria present in the leaves expression the left 7 effectors compared with control. The which suppressed the cell death induced by BAX in *N. benthamiana* leaves were significant higher than that of control leaves (*p* < 0.01). These results demonstrated that the majority of candidate effectors play a role in suppressing host immunity.

### Expression characteristics of candidate effectors regulating plant defenses

**Figure 3:**
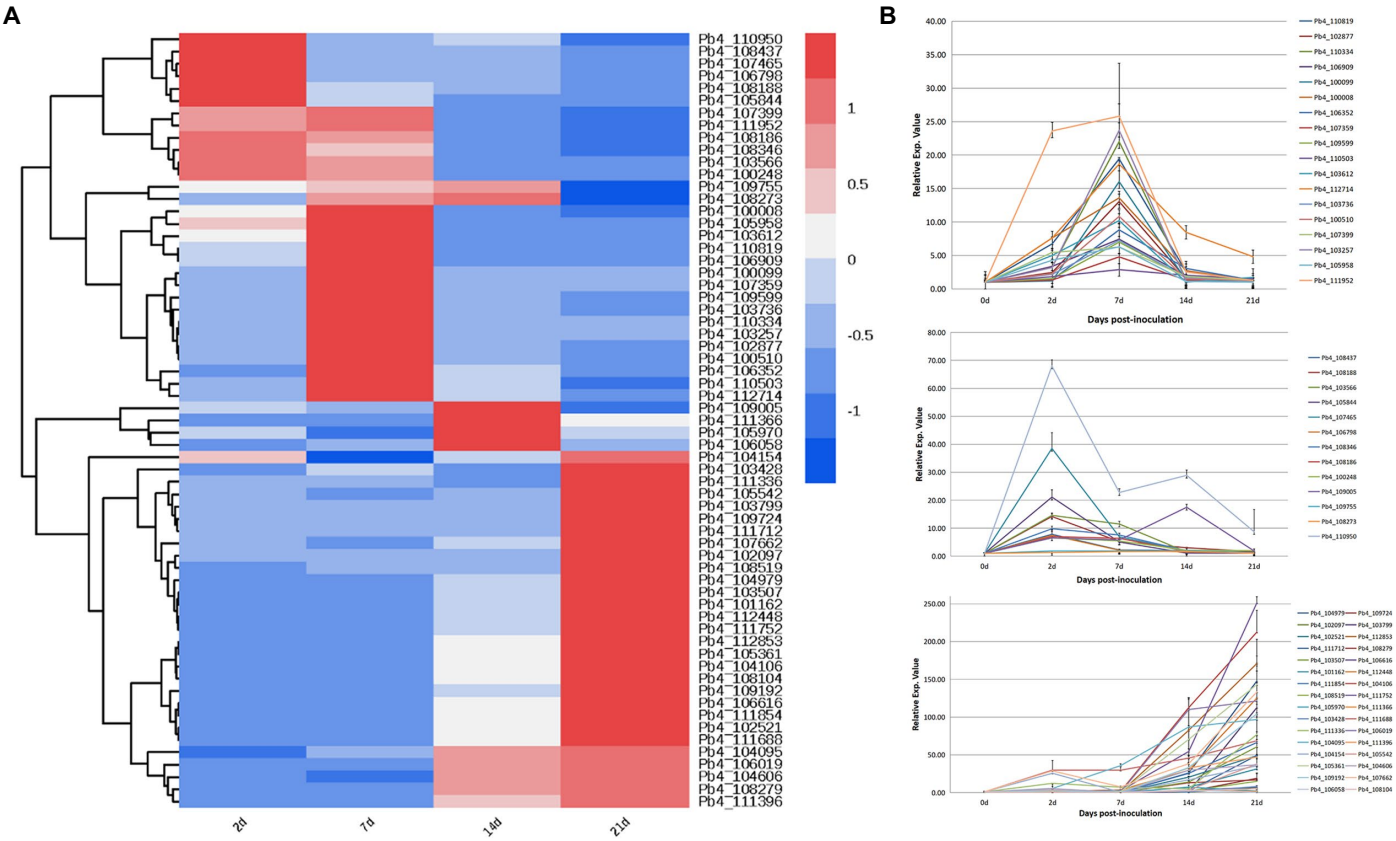
Expression profiles of putative effector genes in the root samples of *Brassica rapa* infected with *P. brassicae*. **(A)** Cluster analysis heat map of all *P. brassicae* effector genes. **(B)** The expression level of these effector genes in different categories.

Transcription levels of the 63 putative effectors were evaluated at 5, 14, 24, and 28 days post inoculation of *B. rapa*. The putative effectors were divided into three categories based on expression pattern (Figure 3A). During primary infection, 13 effector genes showed high expression levels. Of these, 11 continued to decrease up to 28 DPI, except for Pb4_110950 and Pb4_109005. During the second infection, the expression levels of 16 effectors, including Pb_106352, Pb_109599 and Pb_110503, increased significantly at 7 DPI, then gradually decreased after 14 DPI. The majority of effector expression levels, including Pb_102097 and Pb_108104, increased during resting spore formation (after 14 DPI; Figure 3B).

### Subcellular localization of nine RXLR candidate effectors in *Nicothiana benthamiana*

**Figure 4:**
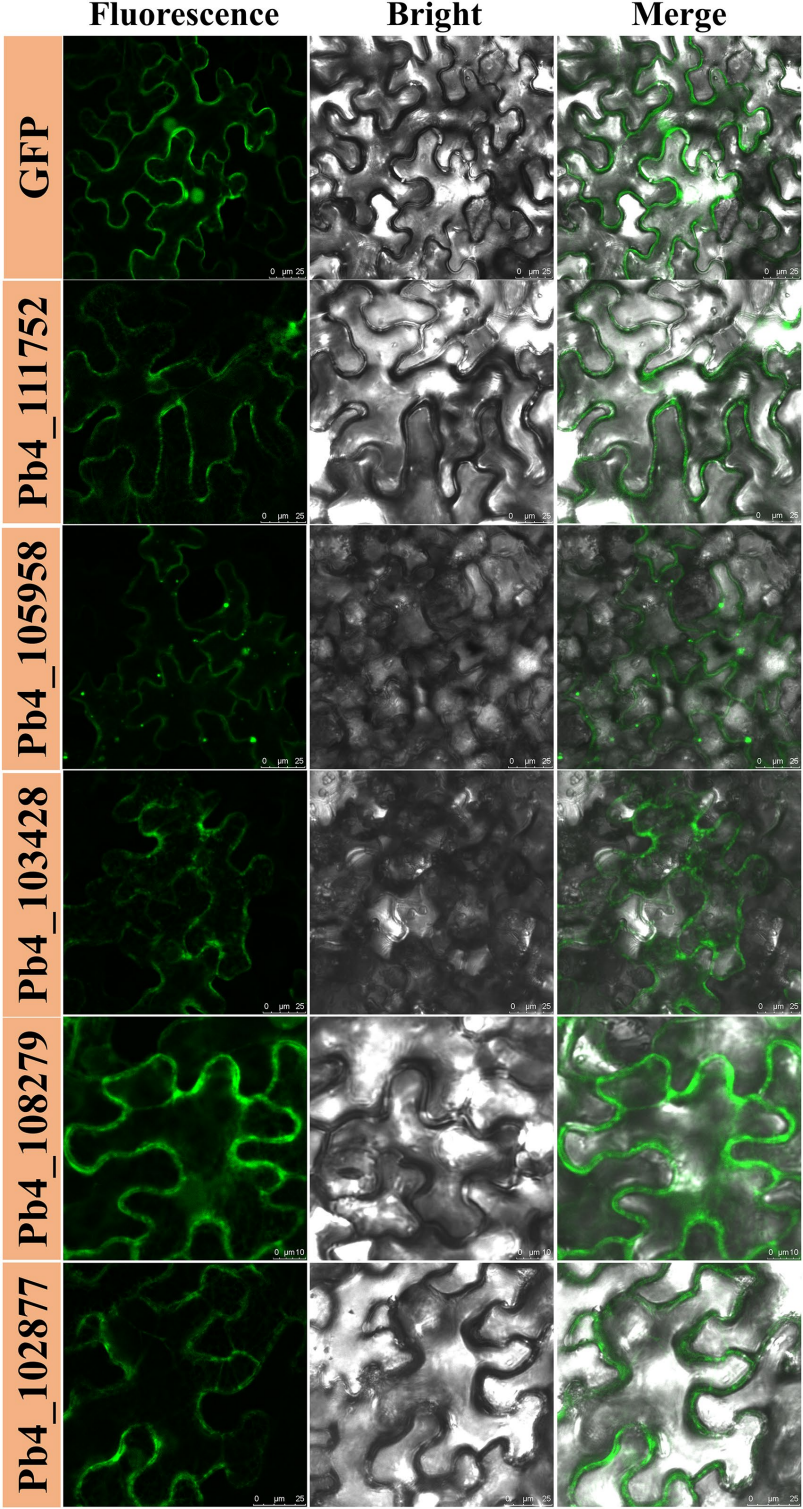
Localization of several candidate effectors with RXLR motif in *Nicothiana benthamiana*.

The localization of pathogen effectors after entering a plant cell could indicate their mode of action (Lamond and Spector, 2003; Spector and Lamond, 2011). To characterize the subcellular localization of five candidate effectors containing RXLR, C-terminal eGFP-tagged constructs were generated and expressed in *N. benthamiana via* agroinfiltration. All these effectors were expressed in the cytoplasm and cytomembrane. The weak green fluorescence signal of Pb4_108279-GFP was also detected in the nucleus. We observed predominant green fluorescence spots of Pb4_105958 dispersed in the cytoplasm, which was probably caused by polymerization of Pb4_105958-GFP proteins (Figure 4). The subcellular localization of these RXLR effectors implied different functions in response to plant immunity.

## Discussion

Identification of effector proteins is key to understanding the interaction between *P. brassicae* and its susceptible host plants. A bioinformatics pipeline approach enables screening of putative effectors based on the genome sequence of *P. brassicae.* However, it is difficult to clarify all functions of the putative effectors identified by bioinformatics. Transient expression assays in the *N. benthamiana* model system employing agro-infiltration have been used to identify effectors in bacteria, oomycetes, fungi and nematodes (Wang et al., 2011; Ali et al., 2015; Fang et al., 2016; Choi et al., 2017). In this study, 518 secretory proteins were screened from the genome of *P. brassicae* strain used in this study. The number of secretory proteins was slightly less than that (558) predicted by Arne (Schwelm et al., 2015). Although, the secretory proteins with special motif, such as RXLR, LXAR and CRN probably function as effectors, some proteins without special candidate motif were also identified in different pathogens (Kemen et al., 2005; Dodds et al., 2009; Malcova et al., 2021). Considering the special relationship between pathogen and host that *P. brassicae* covered by the membrane structure and parasitized in the root cells, there must be active exchange between them, including the secretary proteins. Therefore, the relative broad selection from the putative secretary proteins for function verification was necessary. In this study, 63 putative effectors were cloned based on the bioinformatics analysis of the *P. brassicae* genome. We found that 55 putative effectors could suppress plant immunity triggered by BAX in *N. benthamiana*. And 21 out of 55 could significant suppress the proliferation of Pst DC3000 (*AvrRps4*) in *Arabidopsis. P. brassicae*, an intracellular biotrophic parasite, needs to absorb nutrients from living host cells. Therefore, it secretes a large number of proteins to suppress plant immunity. This phenomenon has also been found in other biotrophic pathogens. Of the 83 putative RXLR proteins of *P. viticola*, 62 could suppress cell death triggered by elicitin in *N. benthamiana* (Xiang et al., 2016). Additionally, 78 of the 95 putative effectors of *H. avenae* could suppress programmed cell death triggered by BAX in *N. benthamiana* (Chen et al., 2013). The universal rule in the biotrophic pathogens probably benefit its propagation and colonization.

Surprisingly, two putative effectors, *Pb4_102097* and *Pb4_108104*, which strongly expressed in the stage of rest spore maturity and release could successfully induce cell death accompanied by H_2_O_2_ accumulation in *N. benthamiana*. These secretary proteins were necessary for the life cycle of *P. brassicae.* The obligate pathogens try their best to hide from host’s immune-response system to hijack the nutrition and populate inside a host (Dimitrios et al., 2015). However, they are waiting an opportunity to escape from host, when their life cycle is coming to end. *Pb4_102097* and *Pb4_108104* showed high expressing level in the stage of rest spore maturity, which probably play an important in facility the rest spore release to the soil.

Many pathogen effectors were screened from secretary proteins identified with agroinfiltration of *Nicotiana benthamiana* method. The best example of plant-specific recognition is the recognition of avirulence effectors either directly or indirectly by nucleotide-binding-leucine-rich-repeat receptors (NLRs), which results in the activation of plant programmed cell death, and cessation of further pathogen growth (Whitham et al., 1994; Tang et al., 1996; Dodds et al., 2004; Rehmany et al., 2005). In *B. rapa*, several clubroot resistant (CR) loci have been mapped, and two resistant genes *CRa*, *Crr1a* are cloned. Both are typical resistance proteins with TIR-NBS-LRR domains (Ueno et al., 2012). Considering the interaction models between other pathogens and hosts, there must be effectors recognized by these CR genes. However, little research on the interaction mechanism between *P. brassicae* effectors and CR genes has been reported. Therefore, it is challenging to determine the interaction mechanism between effectors of *P. brassicae* and clubroot resistant genes.

Effectors delivered into host cells deregulate host immunity in a variety of subcellular compartments (Rovenich et al., 2014). The AvrPiz-t effector of *Magnaporthe oryzae* targets proteasome activity through interaction with the RING E3 ubiquitin ligase APIP6, leading to their mutual degradation and suppression of host immunity (Park et al., 2012). *Hyaloperonospora arabidopsis* directly delivers its effector to the nucleus of the host to interact with the mediator complex that controls interactions between transcription regulators and RNA polymerase (Caillaud et al., 2012). Five of the putative effectors containing the RXLR motif in this study functioned in the cytoplasm of *N. benthamiana.* One effector also functioned in the nucleus. Some RXLR effectors require nuclear localization to trigger or suppress cell death (Du et al., 2015; Xiang et al., 2017; Liu et al., 2018; Qi et al., 2019). Transcriptome analysis revealed a variety of genes responding to *P. brassicae* infection in *B. rapa*. These included the SWEET family responsible for glucose transporter. A large amount of nutrients are required for the propagation of *P. brassicae*, which is probably directly controlled by the effectors delivered into the host nucleus.

In this study, the functions of *P. brassicae* proteins expressed during the different infection stages in the induction and suppression of plant immunity were investigated. Only two putative effectors induced plant immunity and most of the secretory proteins suppressed plant immunity. All the effectors containing the RXLR motif functioned in the cytoplasm and the cytomembrane. These findings improved our understanding of the functions of *P. brassicae* effector interactions between *P. brassicae* and hosts. Whether the effects of these effectors in plant cell death are beneficial for *P. brassicae* infection and the mechanisms by which these effectors manipulate plant immunity are unclear and require further investigation.

## Data availability statement

The original contributions presented in the study are included in the article/Supplementary material, further inquiries can be directed to the corresponding authors.

## Author contributions

ZZ: analyzed the data, performed the experiments, and drafted the manuscript. SL, YY, and SL: contributed to perform the experiments. XL and ZP: conceived the study, participated in its coordination, and helped to draft the manuscript. All authors contributed to the article and approved the submitted version.

## Funding

This project was supported by grants from the China Agriculture Research System (CARS-12).

## Conflict of interest

The authors declare that the research was conducted in the absence of any commercial or financial relationships that could be construed as a potential conflict of interest.

## Publisher’s note

All claims expressed in this article are solely those of the authors and do not necessarily represent those of their affiliated organizations, or those of the publisher, the editors and the reviewers. Any product that may be evaluated in this article, or claim that may be made by its manufacturer, is not guaranteed or endorsed by the publisher.

## Footnotes

^1^ http://www.cbs.dtu.dk/services/SignalP/

^2^ http://pfam.xfam.org/
