# Supplementary figures and images for: Identification and characterization of putative effectors from Plasmodiophora brassicae that suppress or induce cell death in Nicotiana benthamiana
Source: Front Plant Sci. 2022 Sep 20;13:881992. doi: 10.3389/fpls.2022.881992 (PMC9530463; doi:10.3389/fpls.2022.881992)

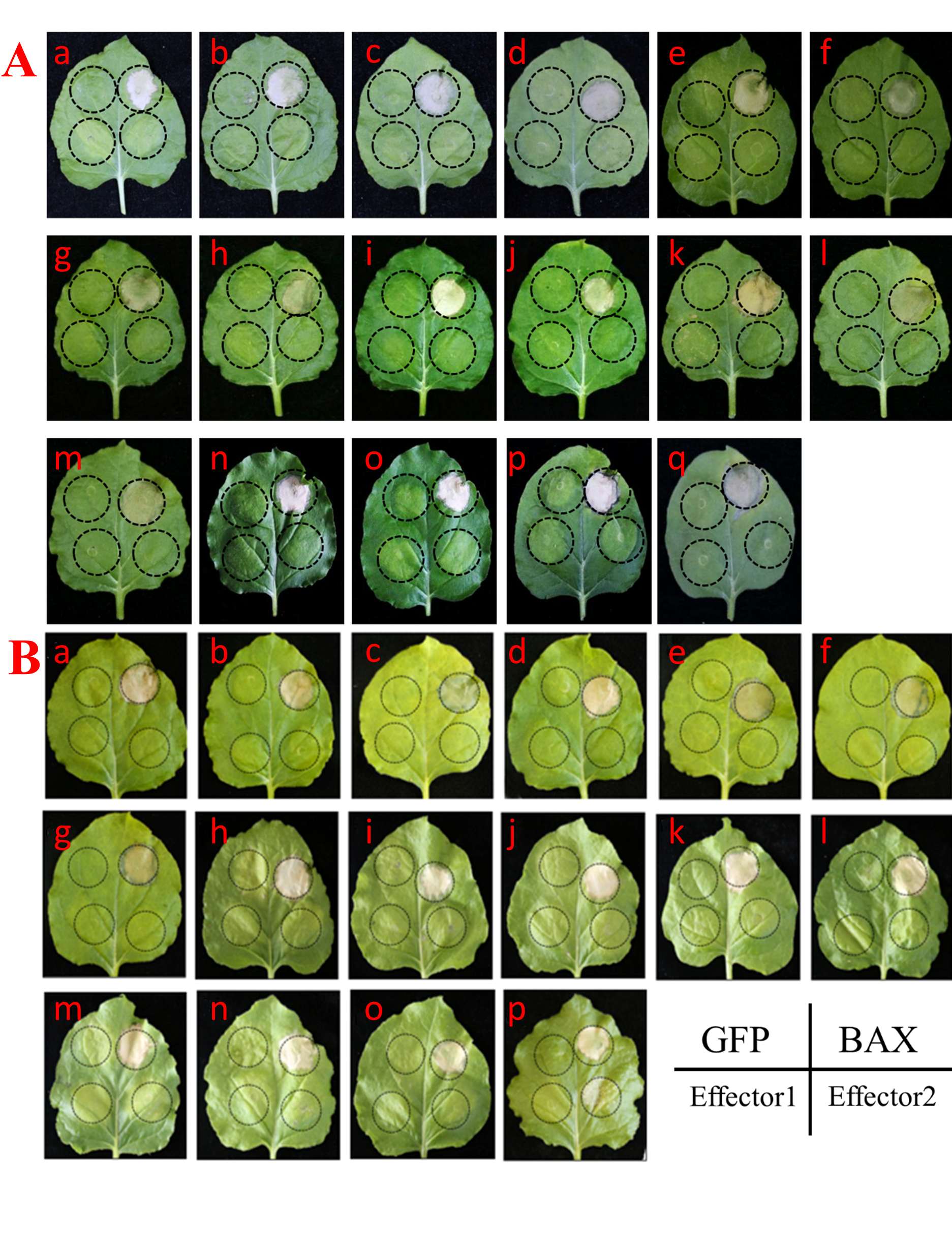

Supplement: Supplementary file 4 [file Image_1.TIF]

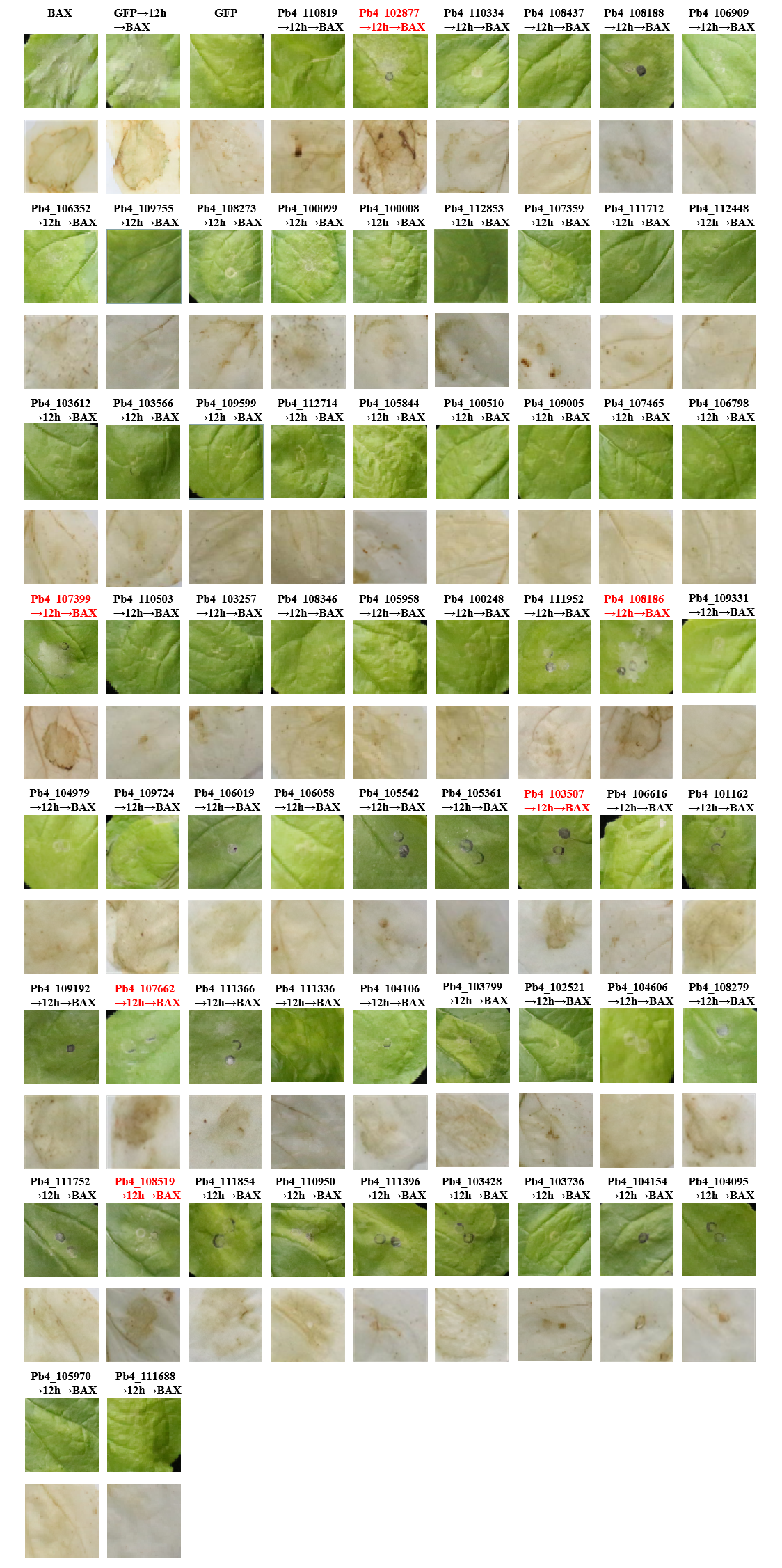

Supplement: Supplementary file 5 [file Image_2.TIF]
